# An Integrated Process and Outcome Evaluation of a Web-Based Communication Tool for Patients With Malignant Lymphoma: Randomized Controlled Trial

**DOI:** 10.2196/jmir.5877

**Published:** 2016-07-29

**Authors:** Inge Renske van Bruinessen, Evelyn M van Weel-Baumgarten, Hans Gouw, Josée M Zijlstra, Sandra van Dulmen

**Affiliations:** ^1^ NIVEL Netherlands Institute for Health Services Research Utrecht Netherlands; ^2^ Radboud University Medical Center Dept of Primary and Community Care Nijmegen Netherlands; ^3^ Hematon Amersfoort Netherlands; ^4^ VU University Medical Center Dept of Haematology Amsterdam Netherlands; ^5^ University College of Southeast Norway, Drammen, Norway Faculty of Health Sciences Drammen Norway

**Keywords:** RCT, communication aid, Web-based intervention, self-help application, hematologic malignancies, lymphoma cancer, patient participation

## Abstract

**Background:**

The complex nature of the medical dialogue and the often emotional context in cancer care present challenges to health care professionals (HCPs) and patients. Patients are increasingly expected to be informed participants and to be able to make conscious decisions, which they often find very difficult. In an attempt to support patients with malignant lymphoma in clinical communication, we developed a stand-alone, Web-based intervention called “PatientTIME.” The development of PatientTIME was based on a participatory intervention mapping framework. Its primary aim is to boost patients’ self-efficacy in patient-professional communication (ie, their confidence when interacting with their HCP). Patients can use this intervention before their hospital visit to prepare for their clinical consultation. PatientTIME is fully automated and use is patient-initiated.

**Objective:**

The aim of this study was to evaluate if and in what way patients benefit from PatientTIME and if it enhances their confidence in clinical communication.

**Methods:**

The intervention was evaluated in a closed randomized controlled trial with continuous recruitment (using online and offline methods to reach potential participants) and data collection. In accordance with the Medical Research Council guidance, we started with a process evaluation. Subsequently, an outcome evaluation was performed focusing on the patients’ perceived confidence in communication with their HCP, measured with the validated PEPPI questionnaire at baseline and at 3 months after participation. Process and outcome data were obtained through Web-based questionnaires, log files (automatically generated files mapping the interactions between program and users), and a logbook (comprising a record of actions and interactions kept by the researchers). Participants were not blinded. A total of 146 patients registered online, of whom 97 gave their informed consent and were assigned at random to the control group (N=34) or 1 of the 2 intervention groups (N=63). Ultimately 87/97 (90%) of these patients actually participated in the study, producing 87 datasets for analysis.

**Results:**

More than half of the intervention group patients reported that the intervention helped them prepare for a clinical consultation; it created awareness about the importance of communication and reinforced their existing communication skills. In the postvisit test, the control group showed a small, nonsignificant improvement in perceived communication efficacy. The intervention group showed a significant improvement in perceived efficacy. However, the interaction effect was not significant, indicating that the improvement solely as a result of the intervention may not be significant.

**Conclusions:**

A considerable number of patients reported that PatientTIME did provide support. We found a trend indicating that in the long run, patients with access to PatientTIME scored better on the perceived efficacy scale than patients without access. However, at this stage we cannot conclude that PatientTIME improves patients’ confidence when interacting with HCPs.

**ClinicalTrial:**

Netherlands National Trial Register (NTR): 3779; http://www.trialregister.nl/trialreg/admin/rctview.asp?TC=3779 (archived by WebCite at http://www.webcitation.org/6iztxJ5Nt)

## Introduction

The interaction between the health care professional (HCP) and the patient is the fundamental vehicle for exchanging information. For the HCP, effective communication is necessary to manage and resolve biomedical and psychosocial problems, which are key issues in cancer care. For the patients it is important to “know and understand,” and the communication serves a purpose in their need to “feel known and understood” [1].

It is important to have effective communication in order to deliver good care. Indirectly, effective communication has been linked to a range of improved patient outcomes such as satisfaction, treatment compliance, perceived quality of life, and physical health [2-6]. However, the complex nature of the medical dialogue and the often emotional context in cancer care are a challenge for HCPs and patients, and the quality of communication often remains suboptimal [7]. Although the HCPs are responsible for the communication process, the increased focus on patient empowerment and shared decision making has broadened the role of patients [8-10]. Patients are increasingly expected to be informed participants and to be able to make conscious decisions [11].

Research shows that such patient participation pays off: if patients participate actively, physicians provide significantly more information overall and respond better to questions [12]. Patients who reach their preferred level of participation experience less anxiety and are more satisfied with the clinical consultation [13]. However, most cancer patients do not achieve their desired level of participation [13,14]. Patients’ communicative contribution appears to be limited [15,16] and patients report unmet communication needs [7,17]. Research has highlighted the importance of not only training the HCPs in communication skills, but also providing cancer patients with support in communication [18-20].

So far, cancer communication studies in clinical settings focus mostly on specific types of cancer, especially breast, prostate, and colorectal cancer [21]. Disease-specific communication instruments are lacking for patients with malignant lymphoma. Via the Dutch patient association Hematon (for leukemia, malignant lymphoma, and stem cell transplantation), these patients have indicated that they often lack the skills needed to be more in control, participate more, and play a more active role during clinical consultations. Research confirms their need for support [22,23]. In an attempt to support patients with malignant lymphoma in communicating with their health professionals, we collaborated with these patients to develop the Web-based intervention “PatientTIME” [24]. Patients can use this stand-alone intervention before their hospital visit to prepare for clinical consultations (see Intervention). The primary aim of the intervention is to positively influence patients’ self-efficacy in patient-professional communication [25,26], that is, their confidence that they can interact with their HCP. Self-efficacy is an important predictor of actual communication behavior [21]. The effectiveness of PatientTIME was tested in a randomized controlled trial (RCT) with self-efficacy as the primary outcome measure.

Randomized controlled trials are considered to be the most rigorous way of evaluating effectiveness in the medical context. Traditionally, the main focus is on reporting prespecified outcomes. This evaluation method is predominantly applied in interventions with one active variable, for example, the effect of a drug on survival [27]. In interventions like PatientTIME, different active ingredients (Table 1) are combined and evaluated simultaneously. Oakley et al [28] argue that when evaluating such a “complex” intervention, incorporating a process evaluation would support and improve the interpretation of outcomes. Process evaluations look into the nature of the intervention, how it is delivered, and what actually happens during the intervention [29,30]. It can improve the validity and interpretation of outcomes, help refine the intervention, and provide necessary information for replication [27,30]. Despite the rise of complex interventions, few studies combine process *and* outcome evaluations.

In this study, knowledge about the process characteristics is expected to help in improving the PatientTIME intervention: it may show how to reach different patient groups and it can support the right interpretation of outcomes. Moreover, the process evaluation provides the context in which the data for the outcome evaluation are gathered. The main question to be answered by the outcome evaluation is “Does the intervention increase participants’ confidence in interacting with their HCP?”

The ultimate goal is to implement PatientTIME as a publicly available, stand-alone intervention, that is, without the research context and without the involvement of professionals. In addition to giving insight into the effectiveness, the results of the study can help us optimize PatientTIME as a stand-alone intervention.

## Methods

### Procedure and Ethical Approval

In accordance with the Medical Research Council (MRC) guidance, we started with a process evaluation focusing on the reach of the intervention and the extent to which it was used as intended [31]. Subsequently, the outcome evaluation was performed, focusing on the patients’ perceived confidence when interacting with their HCP. The research ethics committee of the Radboud University Nijmegen Medical Centre evaluated the RCT protocol and concluded that the study did not fall within the remit of the Dutch Medical Research Involving Human Subjects Act (WMO). The study is registered in the Netherlands National Trial Register (trial registration number NTR3779). Written informed consent forms were used.

### Intervention

**Figure 1:**
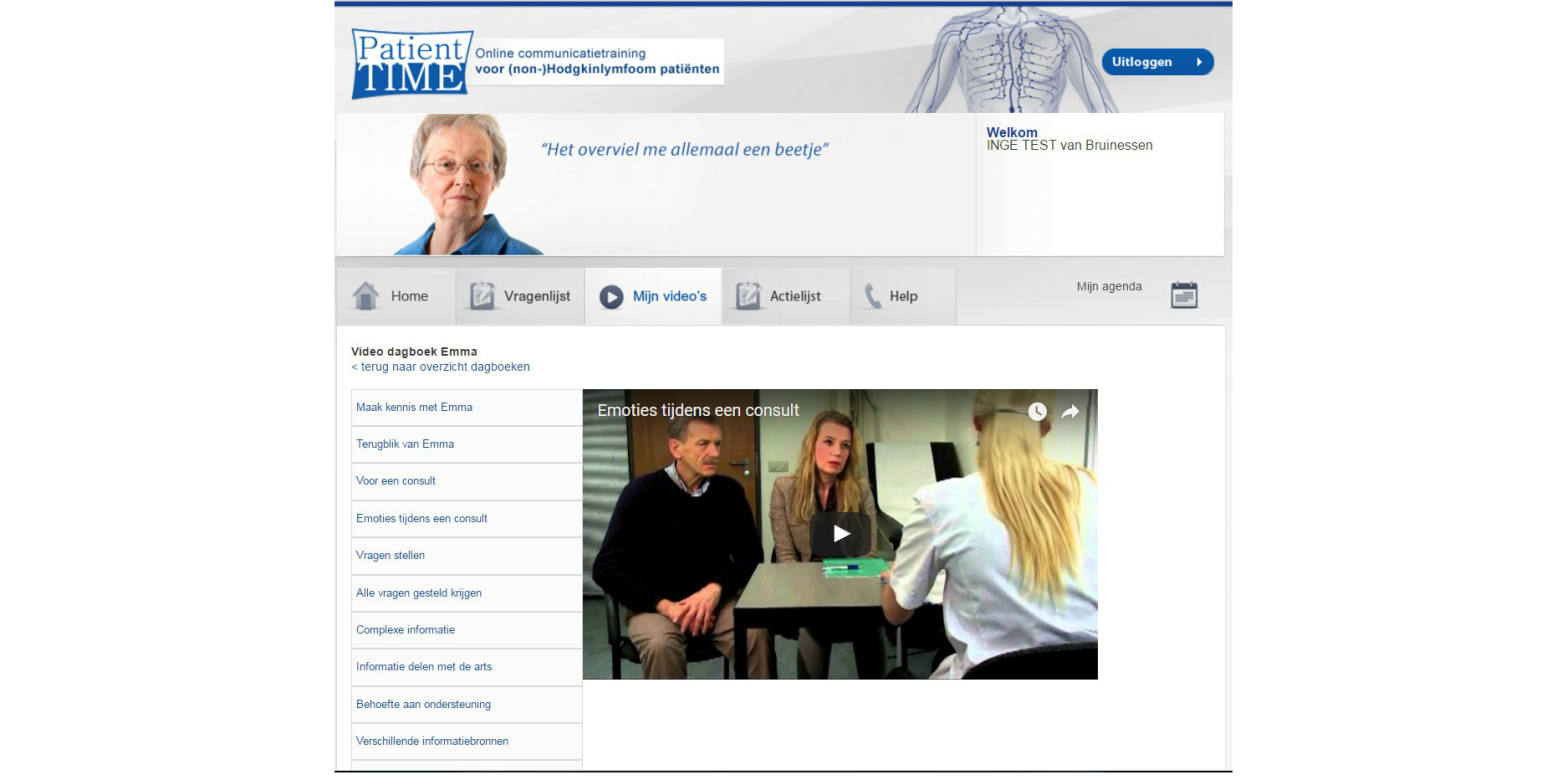
Screenshot of the PatientTIME intervention.

The Web-based PatientTIME intervention aims to support patients in gaining more control over the communication with their HCP. The intervention development was guided by the intervention mapping framework applied in close collaboration with patients [24] and makes use of different theory-based methods: modeling, tailoring information, previsit goal setting, and listening to visit recordings. The central source of information in the intervention consists of 58 short video fragments (47-180 seconds) showing simulated patients demonstrating different communication skills during medical encounters (eg, stating the need for support, dealing with emotions, or asking questions; Figure 1) [32]. The fragments are based on communication barriers identified by the targeted population in a previous study [22]. A question prompt sheet (QPS) and an option to replay an audio recording of the user’s hospital visit were also included in the intervention. The functionality and intended use of these individual components are described in Table 1. The collaborative partners (2 hospitals, the patient association, the funding organizations, and a research institution) were listed on an information page.

**Table 1:** The intended use of the individual intervention components.

Intervention component	Intended use
Video library	Before a clinical consultation, a subset is selected from the 58 video fragments available for use in the video library. The selection is tailored to the user’s preferences and needs at that time and stored in the user’s personal video library. When the intervention is used again, new video fragments are added to the library along with the previously viewed videos (which are still available for viewing). Per consultation, video clips regarding a maximum of 3 communication themes are provided (6 clips in total). When using the intervention for the first time, a maximum of 4 introductory clips are added to the theme clips.
Question prompt	A prompt was integrated to encourage patients to set goals and prepare questions before the consultation. Patients can also formulate questions or remarks while watching the video fragments. The question prompt sheet can be printed or emailed to the patient’s address.
Listening back to the consultation	A consultation audio recording can be uploaded, attached to the consultation date in the agenda, and replayed at any time.

### Recruitment

PatientTIME is a stand-alone intervention; that is, patients can register and use the intervention without referral or the involvement of a professional. Several online and offline actions were organized to create awareness of the availability of PatientTIME: (1) spreading leaflets at hospital waiting rooms, (2) giving short presentations during patient information meetings arranged by Hematon and at hospitals, (3) short news flashes in Hematon’s digital newsletter and magazine, (4) distributing our own project newsletters, and (5) using social media (Twitter, forums). Additional actions were initiated during the inclusion period: (6) we sent information packages and leaflets to hematologists asking them to distribute the information among their patients, and (7) we sent short news flashes about the study to local newspapers. Patients could express their interest in participating via registration on the PatientTIME website [21] between March 2013 and May 2015. After registration, the study’s inclusion criteria were verified. Patients were eligible for participation if they were aged 18 years or older, had been diagnosed with malignant lymphoma, had at least one (follow-up) consultation with their HCP per year, had a good understanding of the Dutch language, were receiving treatment or follow-up care in a Dutch hospital, and had access to a computer with an Internet connection.

If the inclusion criteria were met, patients received a consent form with additional information by post. As soon as they had returned the informed consent form, their account was activated and an email was automatically sent to the patient with log-in details. If no consent form was returned, the patient was reminded by email after 2 weeks and by phone 1 week after that.

### Design

The intervention was implemented in the form of a 3-armed RCT with continuous recruitment and data collection. The computer assigned participants randomly to 1 of 3 groups: (1) a control group, (2) an intervention group, and (3) an intervention audio group. Software that enabled blocked randomization (block size 3) was used to balance the groups.

**Figure 2:**
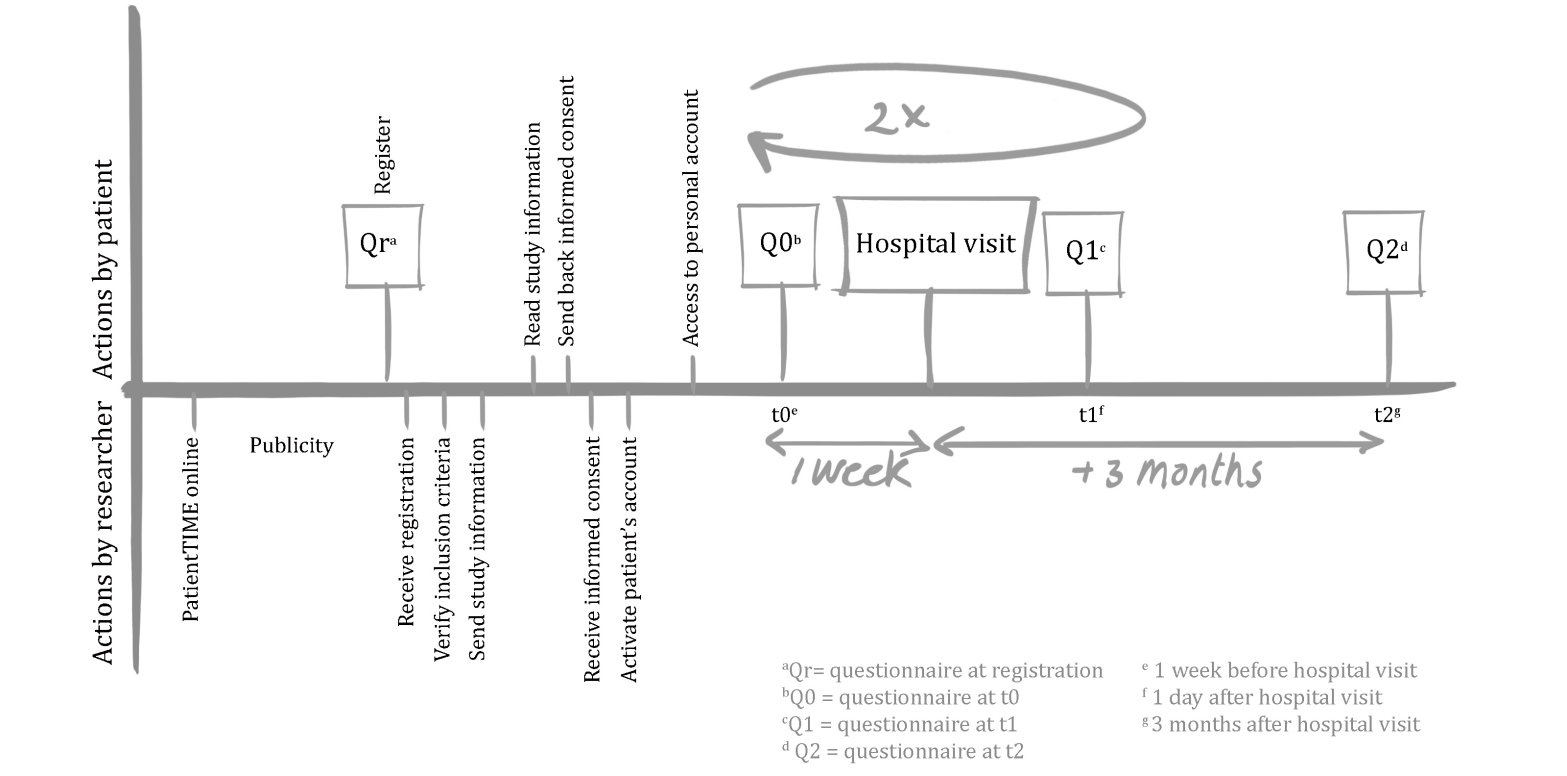
Timeline randomized controlled trial steps.

The 2 intervention groups differed in one component: having or not having the opportunity to listen afterward to the audio recording of their clinical consultation. For the purpose of this outcome evaluation, the 2 intervention groups were evaluated as 1 group and compared with the control group. We made this decision for practical reasons. Patients who had the opportunity to listen afterward to their consultation generally did this after completing the postvisit questionnaire (Figure 2). Therefore, the experience of replaying and listening to the consultation could not have influenced their answers. Moreover, some patients in the intervention group where we did not actively encourage them to record their consultations (group 2) still made recordings on their own initiative.

**Figure 3:**
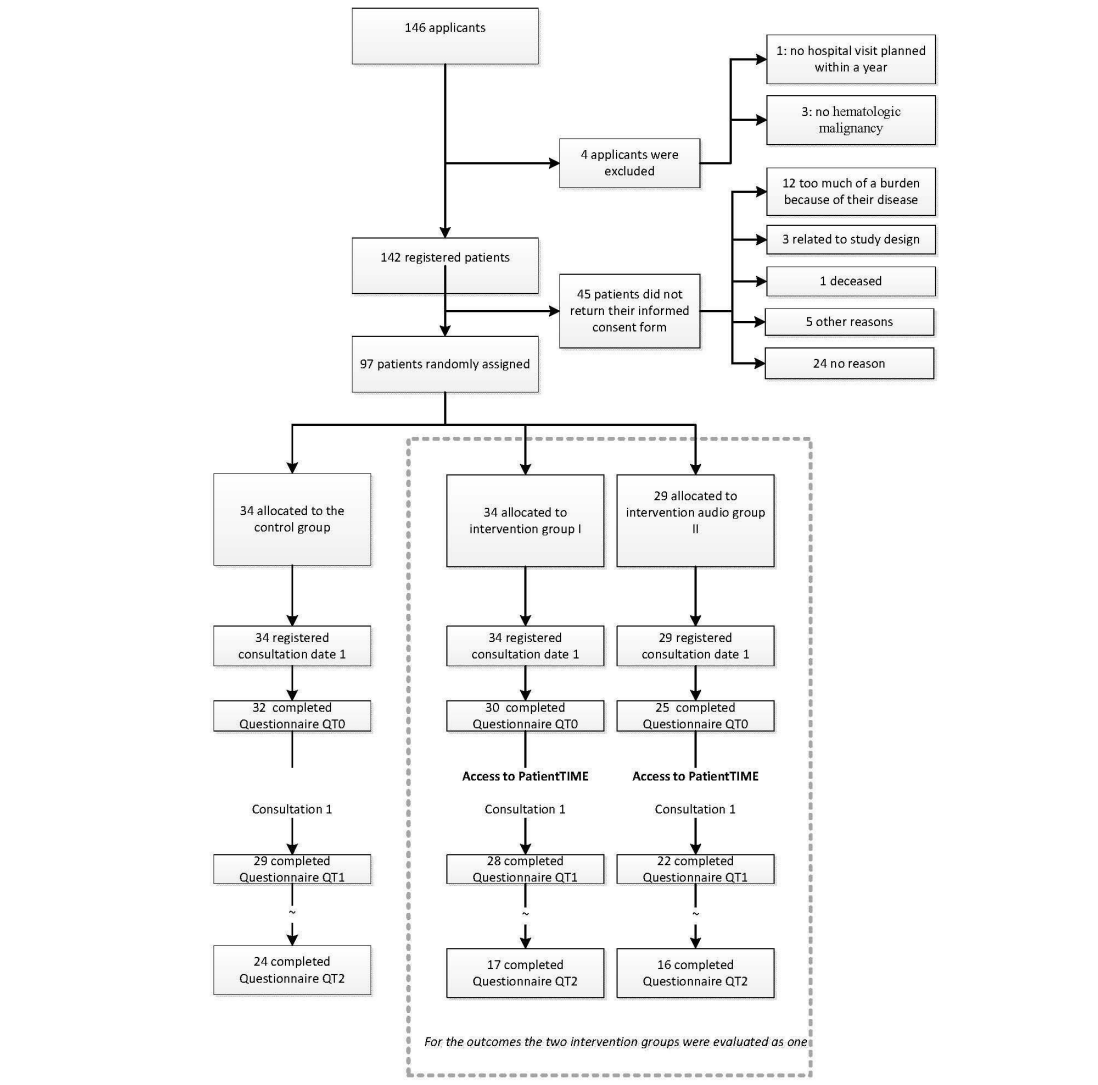
Flowchart.

All participants were given access to a personal secure Web account with questionnaires and an integrated digital agenda. The agenda had an automatic reminder system. This system led them through the study with personalized messages and questionnaires before and after clinical consultations (Figure 2). The date of the participant’s forthcoming hospital visit determined the start of his or her participation. One week before this visit, participants received an automatic email reminder with the request to complete the previsit questionnaire on their Web account. After having completed the previsit questionnaire, the control group received no further information. The 2 intervention groups were given access to the intervention content (Figure 3). The selection of video fragments taken from the video library was based on the participant’s self-reported previsit communication preferences and needs. The intervention audio group participants were also instructed—if permitted by their HCP—to record their consultations, upload the audio recordings, and listen afterward to them. The recordings were also available to the researchers for analyses. We provided these participants with a small audio-recording device and extra information about consultation audio recordings.

The day after the consultation, patients received an automatic reminder with a link to the postvisit questionnaire. If a second and third consultation was planned within the participation year, the cycle was repeated with less comprehensive questionnaires. If necessary, a maximum of 2 reminders were sent by the researcher reminding the participant to complete the questionnaires or register a consultation date. The control group had access to the educational content of the intervention after participation.

There was no downtime during the evaluation period. PatientTIME’s content and functionality were frozen for the duration of the trial. Necessary bug fixes were made relating to questionnaires that were not always directly available after the clinical consultation.

Participants were not blinded as they could see whether they had access to the content of PatientTIME or not. As self-confidence in communication was the primary outcome measure, the power calculation was based on the primary outcome measure of the RCT, the PEPPI (Perceived Efficacy in Patient-Physician Interactions) questionnaire (see Data Collection and Measures) [26]. We expected the intervention to result in a decrease of 5 points on the sum score (reflecting higher confidence), which corresponds to an effect size of 0.70. To detect this difference with a power of .80 and alpha of .05, we needed 50 patients in the intervention group and 50 patients in the control group.

### Data Collection and Measures

The process and outcome data were measured through (1) Web-based questionnaires (Qr, Q0, Q1, and Q2; see Figure 2), (2) log files, that is, automatically generated files mapping the interactions between the program and its users, and (3) a logbook, comprising a record of actions and interactions that was kept by the researchers.

According to the MRC guidance, the selection of dimensions that are evaluated should be adjusted to the intervention under study. A total of 7 process dimensions were selected that may influence outcome measures in this study (Table 2).

**Table 2:** Dimensions of the PatientTIME process evaluation.

Dimensions	Purpose	Research questions (data source)
**Delivery**		
Uptake	Insight into the procedures used to reach users, and information on who was reached	Were the publicity actions conducted as planned? (Logbook) Who showed interest in participating? (Questionnaire Qr) What were the reasons for not participating? (Logbook)
“Dose” delivered	Insight into the exposure to the intervention	To whom was the intervention delivered, and how does this compare to the population of interested patients? (Questionnaire Qr)
Participation rate	Insight into the characteristics of participants^a^ and their reasons for participating	Who participated in the evaluation, compared with the total group who were invited to participate in the intervention? (Questionnaire Qr, Q0)
Fidelity	Insight into the extent to which the intervention was provided as planned	Which adaptions made to the intervention for the benefit of the effect evaluation may have influenced use and outcomes? (Logbook) What was the time between registration and the consultation date? (Questionnaire Qr) To what extent were reminders necessary for the benefit of the effect evaluation? (Logbook)
**User-program interaction**		
Dose received (attrition)	Insight into the actual exposure to the intervention and the use of its different components	How many video fragments were watched per patient? (Log files) How many patients used the question prompt sheet? (Log files) How many patients audio-recorded their consultation? (Log files)
Usability	Insight into the usability of the intervention	How was the satisfaction with the intervention rated? (Questionnaire Q1)^b^
**Contextual factors**		
Patient’s context	Insight into the contextual factors at the micro level	What influence does the patients’ context have and how could the context have influenced the outcome evaluation? (Logbook)

^a^ A patient was defined as a “participant” if he or she registered the first consultation date and completed the previsit questionnaire Q0.

^b^ The usability was evaluated with the System Usability Scale, a 10-item questionnaire that gives an overview of satisfaction with the program, resulting in a sum score (range 0-100) [33]. Usability tests had already been conducted with patients on a small scale during the development phase. However, as usability is strongly associated with use, attrition, and dropout, we decided to measure it on a larger scale as well.

In addition to the dimensions taken from the MRC guidance, the intervention group was asked if and in what way the intervention had helped them. All “evaluative” responses (answers to the question) were coded as “positive” or “negative.” To prevent subjective interpretation of the data, 2 researchers coded all fragments. The interrater reliability was good (81% agreement). Disagreements were discussed to come to an agreement.

For the primary outcome measure, participants were asked to complete the validated, 10-item PEPPI questionnaire twice: at t0 and t2. The PEPPI questions all begin with “How confident are you in your ability to...” and assess a patient’s confidence in their ability to communicate with their HCP on a 5-point Likert scale (1 = very confident to 5 = not confident at all) [26,34]. Item scores were summed (giving a range of 10-50), whereby lower scores reflect higher self-efficacy. The PEPPI questions used at t1 were reformulated so that they could also be used as a postvisit scale, measuring efficacy experienced directly after the visit.

### Statistical Analysis

Descriptive statistics were used to characterize the sample and to analyze the process measures.

Differences between groups were analyzed using *t* tests and chi-square tests, where appropriate. To analyze short-term effects, linear regressions were conducted to test for differences in experienced efficacy measured at t1 between the control and intervention groups, adjusting for the previsit PEPPI score. To analyze long-term effects, multilevel regressions were conducted to test for differences on the PEPPI scale between the control and intervention groups at t2 and t0. The analysis of the open questions was performed using MAXQDA 11 software [35]. Other process and outcome data were analyzed with Stata 13.

## Results

### Participant Flow

A total of 146 potential participants registered (*uptake*) of which 97/142 (68%) patients met the inclusion criteria and signed the informed consent form (*dose delivered*; Figure 3). This group included 5 patients who were not diagnosed with malignant lymphoma but with a hematologic malignancy with a comparable impact (eg, chronic lymphocytic leukemia or multiple myeloma).

Of the 49 potential participants who were not included, 4 were excluded by the researchers. The remaining 45 did meet the inclusion criteria but did not return the informed consent form. Compared with these 45 patients, the 97 participants had a significantly higher level of education, were more likely to be a member of a patient organization, had a longer elapsed time between diagnosis and registration, and were more likely to have had or started treatment (Table 3).

All 97 patients in the RCT registered their first consultation date, which was a precondition for being sent the previsit questionnaire (Q0). Subsequently, the *participation rate* was 90% as 87/97 patients completed Q0 and were marked as participants. Compared with the nonparticipants (N=10), the 87 participants had a higher level of education and used the Internet on a more regular basis. After having completed Q1, a total of 76 participants registered a second consultation date and 52 participants a third consultation date as well. There was no significant difference between the control and intervention groups in the proportion registering multiple consultations. Because not every participant had a second or third consultation planned within the participation time span (1 year after registration), the analysis of the outcomes is based on the first consultation only.

### Process Evaluation

The identified process results that facilitated correct interpretation of the outcomes were part of the delivery domain (eg, fidelity, reach; see Table 2) and user-program interaction domain (eg, exposure, use of different components; see Table 2), in addition to the benefits perceived by the patients.

**Table 3:** Background characteristics and outcomes.

Variable	Value	Registered	Randomized	Participant	Control	Intervention
		N=142	N=97	N=87	N=32	N=55
Age (at registration)	Mean in years (SD)	57 (14)	55 (13)	56 (13)	56 (14)	55 (13)
Sex, n(%)	Male	63 (44)	40 (41)	34 (39)	14 (44)	20 (36)
Education^a^, n(%)	Low	16 (11)	6 (6)	2 (2)	2 (6)	0
	Medium	39 (27)	23 (24)	19 (22)	7 (22)	12 (22)
	High	89 (61)	68 (70)	66 (76)	23 (72)	43 (78)
Member of a patient organization^b^, n(%)	Yes	71 (50)	60 (62)	56 (64)	16 (50)	40 (73)
	No	70 (49)	37 (38)	31 (36)	16 (50)	15 (27)
Internet use^c^, n(%)	Daily	128 (90)	90 (93)	90 (93)	29 (91)	52 (95)
	Weekly	10 (7)	6 (6)	6 (7)	3 (9)	3 (5)
	Monthly	2 (1)	1 (1)	0	0	0
**Disease-related information**						
Diagnosis, n(%)	Hodgkin lymphoma	31 (22)	22 (23)	20 (23)	5 (16)	15 (27)
	Non-Hodgkin lymphoma	105 (74)	70 (72)	63 (72)	26 (82)	37 (67)
	Other	6 (4)	5 (5)	4 (5)	1 (3)	3 (5)
Type^b^, n(%)	Indolent	57 (40)	38 (39)	35 (40)	13 (41)	22 (40)
	Aggressive	41 (29)	26 (27)	23 (26)	8 (25)	15 (27)
	Combination	13 (9)	10 (10)	8 (9)	4 (13)	4 (7)
	Not known (yet)	30 (21)	23 (24)	21 (24)	7 (22)	14 (25)
Time elapsed since diagnosis	Mean in years (SD)	5.4 (7.7)^c^	6.5 (8.3)^b^	6.2 (5.5)^b^	3.2 (3.7)	8.0 (10.0)
	< 1 year, n(%)	42 (30)	24 (25)			
	> 1 year, n(%)	100 70)	73 (75)			
Treated, n(%)	Yes	121 (86)	89 (92)	79 (91)	4 (13)	4 (7)
	No	20 (14)	8 (8)	8 (9)	28 (88)	51 (93)
Current status^b^, n(%)	Awaiting test results or treatment (plan)	19 (13)	9 (9)	9 (10)	4 (13)	5 (9)
	Currently getting treatment	33 (32)	22 (23)	19 (22)	8 (25)	11 (20)
	Wait-and-see	36 (26)	26 (27)	25 (29)	9 (28)	16 (29)
	Remission	53 (38)	40 (41)	34 (39)	11 (34)	23 (42)
**Confidence in communication ** **(PEPPI^d^)**						
Perceived, previsit (t0)	Range 10-50 (Cronbach alpha .94)	N/A^e^	N/A	20.5 (6.3)	20.3 (6.0)	20.7 (6.4)
Experienced, postvisit^f^ (t1)	Range 10-50 (Cronbach alpha .93)	N/A	N/A	16.5 (7.8)	15.9 (6.7)	16.9 (8.3)
Perceived after > 3 months^g^ (t2)	Range 10-50 (Cronbach alpha .93)	N/A	N/A	19.3 (5.4)	19.2 (6.4)	19.3 (4.7)

^a^ Classification according to the guidelines of Statistics Netherlands.

^b^ One missing.

^c^ Two missing.

^d^ PEPPI: Perceived Efficacy in Patient-Physician Interactions.

^e^ N/A: not applicable.

^f^ Ntotal=78, Ncontrol=28, Nintervention=50.

^g^ Ntotal=57, Ncontrol=24, Nintervention=33.

Regarding the delivery domain, we concluded that the publicity actions were conducted as planned. On the whole, the intervention was indeed used as a stand-alone program; most participants did not need any help or reminders from the researchers. The questions they asked (mainly by email) concerned problems with logging in or study questionnaires that were not yet accessible. For the first consultation, 18 of the 97 randomized patients received at least one reminder to complete their previsit questionnaire.

We noted one issue regarding the “fidelity” that may have influenced outcomes. At registration, patients had to provide the date of their upcoming consultation. Of the potential participants who did this, 44/138 (32%) had their consultation planned within a fortnight. These patients were less likely to participate in the study (59%) compared with the patients who had their consultation later (76%).

Regarding the user-program interaction domain, we report the actual use of the different intervention components that may have influenced the outcomes. Of the participants assigned to the intervention group, 55/63 (87%) completed Q0 and had access to PatientTIME. This group viewed on average 6 (SD 3.5) of the 10 selected video fragments before their first consultation. Of all the fragments they watched, 76% were viewed from beginning to end. The QPS was used to write down questions, physical complaints, and concerns by 20/55 (36%) of the participants who had access to it. Of the intervention audio group 9/29 (31%) participants reported after consultation that they had recorded their visit, 13/29 (45%) reported that they had not, and 7/29 (24%) did not complete this question. Of the participants who did not record their consultation, 8/13 said that they decided that they did not want to make the recording, 2/13 said the HCP did not give permission, 2/13 said they did not want to ask the HCP and 1/13 forgot the recording device.

Regarding the “usability,” 50 intervention group participants completed the System Usability Scale questionnaire at Q1, revealing an average satisfaction score of 73 points (SD 16) on the 0-100 scale, which is considered “good” according to the study by Bangor et al [36].

As for perceived benefits, in the intervention group, 46/50 (92%) participants completed the open question “Did you benefit from the website?” at t1. The positively coded answers (59%) explained that (1) the website was instructive, insightful, or helpful for the preparation of a clinical consultation; (2) the website created awareness about the importance and role of communication; and (3) participants recognized the scenarios, which gave them the feeling that they were doing all right.

I’ve been thinking somewhat more about the questions I was going to ask the medical specialist.

It makes you more aware of yourself, but also of the doctor’s role.

The negatively coded answers (41%) referred to (1) bugs in the questionnaire or usability problems in the website and (2) the fact that the information did not add anything to what was already known or experienced.

Not that much. I have the idea that I am already quite outspoken during the consultations with my oncologists.

Not much because I have a good relationship with my physician. I can discuss everything.

### Outcome Evaluation

The results on the PEPPI scale at t0 and t2 (Table 3) were skewed to the lower scores, reflecting a group of patients with a high level of self-confidence about interacting with their HCP. The same skewed trend was visible at t1, reflecting a group of participants who were well able to reach their goals.

There were no differences on the PEPPI scale between the control and intervention groups at baseline (t0, *P*=.78). Directly after the consultation (t1), there was no difference between the control and intervention groups in the way they experienced their efficacy during the consultation (controlling for the previsit PEPPI score). The results remained the same when controlling for sex, the level of education, and time since diagnosis.

Comparing the PEPPI score at t2 with the PEPPI score at t0, the control group showed a small improvement in the level of perceived efficacy. This improvement (−0.38 points) was not significant (*P*=.69). The intervention group also showed an improved level of perceived efficacy. This improvement (−1.97 points) was significant (*P*=.02). The interaction effect (−1.59), however, was not significant (*P*=.20), indicating that the improvement solely as a result of the intervention may not be significant. In addition to this primary analysis, we did not find an association between the PEPPI outcomes and the number of video fragments watched.

## Discussion

### Overview

In this paper, we described an integrated process and outcome evaluation of the Web-based intervention PatientTIME. PatientTIME was developed with the aim of helping patients with their communication during clinical consultations. With the process evaluation, we aimed to sketch the context in which the outcome evaluation was performed. The process evaluation was also used to obtain input on how to improve the intervention and its reach before making the intervention publicly available. In the outcome evaluation, we focused on the patients’ perceived confidence in interacting with their HCP.

### The Process Evaluation

As a result of the process evaluation, we identified a substantial number of delivery and interaction aspects that will help to improve the functionality of PatientTIME and its reach. Looking at the delivery, we want to highlight the recruitment process. The chosen method may resemble how patients are informed in practice about publicly available Web-based interventions, but despite the various publicity actions during the recruitment period, this approach did not appear to be enough to recruit a large, diverse group of patients for the study (see also Trial Participants section).

The patient-program interaction evaluation showed how the different PatientTIME elements (video fragments, QPS, and consultation audio recordings) were used. The core content of the intervention, the video fragments, was well used. The percentage of fragments viewed entirely (76%) may even be an underestimate (fragments were only logged as “ended” when the screen switched back to start).

The QPS was used less frequently, and slightly less compared with other studies that evaluated the use of open QPS tools in oncology care [37,38]. We do not know if the limited use was due to the design of the QPS, the fact that patients found it difficult to articulate questions, or the fact that patients preferred to make their notes on paper. Albada et al found that patients need to be motivated to use a QPS. Information about why and how to use the QPS could have been made more explicit in the PatientTIME intervention [37]. In the intervention audio group, more than half of the participants did not record their consultation on audio. There appeared to be a variety of obstacles. Other studies show that cancer patients highly value consultation audio recordings and that the majority benefit from listening to the consultation afterward [39,40]. However, in these studies the HCP facilitated the recording, whereas in our study patients were encouraged to take the initiative. We may have failed to provide sufficient information to remove existing obstacles, or the fact that it was part of a trial may have caused patients to be reluctant. To conclude, the QPS and audio recording options should be improved in the PatientTIME intervention.

More than half of the intervention group patients explained that the intervention helped them prepare for a clinical consultation, created awareness, and/or reinforced their existing communication skills. Patients who reported no benefit explained that they already had a good understanding with their HCP, had a lot of experience, or considered themselves as sufficiently participative and empowered already.

### The Outcome Evaluation

Looking at the data on confidence in interaction, we found a trend indicating that in the long run, patients with access to PatientTIME scored better on the perceived efficacy scale than patients without access. Differences were, however, small and we did not reach our aim of an improvement of 5 points. Therefore, we cannot conclude at this stage that the intervention positively influenced participants’ confidence in their ability to talk with their health professionals.

### The Results in Perspective

The strengths and limitations related to the study and intervention put the results in perspective and illustrate our lessons learned. We identified four important aspects.

#### Trial Participants

The participants were mainly more educated, experienced patients who were already quite confident in their ability to talk with HCPs. It is a well-known phenomenon in eHealth research that patients with a high level of education are overrepresented in these studies [41,42]. However, a recent study in the Netherlands revealed that the majority of health care users are using the Internet to get information about care and health [43]. This makes it reasonable to assume that a broader group will find PatientTIME when it becomes publicly available. For the outcome evaluation, it is therefore a limitation that less experienced patients (patients who had only recently been diagnosed) and patients with a low initial confidence in their ability to interact were not well represented.

The possible explanations for this are (1) we did not reach them, (2) the idea of a communication support tool did not appeal to them, or (3) they did not want to be involved in a research project. Insight into the reach of the intervention is limited because of the lack of information about patients who read about the intervention but decided not to register for participation. The second explanation seems unlikely given the findings of a recent study among patients with a chronic illness (including cancer patients) that revealed that a considerable number (39%) are interested in communication support [44]. Participating in a study like this demands much more from patients than only using the PatientTIME intervention. We received many questions about the consultation audio recording in particular and patients had concerns about this component. All these extra elements may have made patients reluctant to take part.

#### Outcome Measure

The participants in this study appeared to be highly confident in their ability to talk with the HCP at baseline, which left little room for improvement (ceiling effect). On the one hand, this suggests that we reached a group who largely did not need support in communication. On the other hand, these patients did enroll for the study and the core information in the intervention tool was well used. This indicates that these patients were interested in improving their communication skills. Perhaps they saw a mismatch between their preferred role and their behavior in the consultation room. This casts doubts on our decision to measure effectiveness with the PEPPI questionnaire only. Bensing et al reported that patients’ behaviors in the consulting room are not necessarily a reflection of their self-reported preferred behavior [45]. Because we did not observe the consultations, we cannot describe participants’ actual behavior or how PatientTIME may have influenced this. Although the PEPPI questionnaire is a good measure for tracking confidence in medical communication, observations of actual communicative behavior can be a valuable addition. Moreover, the lack of power limits the usefulness of the PEPPI results, as the control group did not reach the required 50 participants. It would also have been interesting to have the patients complete the PEPPI questionnaire again immediately after viewing the PatientTIME content, to measure if their level of confidence had changed as an immediate result.

#### The One-Sided Intervention Approach

PatientTIME was solely focused on patients, without the interference of or intervening in the communication style of the HCPs. Butow et al found that their patient-targeted communication intervention was only effective when the patient consulted a clinician who endorsed the intervention [46]. The one-sided approach used in PatientTIME may have decreased the potential reach and effect.

#### Implicit Trial Effects

Two trial effects were identified that may have influenced outcomes. First, regardless of whether participants were allocated to the intervention group or the control group, it is likely that the mere fact of participating in a communication study highlighted the importance of the participant’s role in medical communication. Increased awareness of being observed in a trial setting may have affected outcomes (pre- and postvisit), which limits generalizability (Hawthorne effects).

Second, the relatively lengthy time between registration and getting access to the intervention tool (because study information needed to be provided and informed consent obtained) delayed the inclusion process. This may have excluded patients who were looking for communication support shortly before their planned consultation. Ideally, access should be given immediately.

### Conclusions

A considerable number of patients indicated that the intervention was helpful in preparing for a clinical consultation, created awareness about the importance of communication, and reinforced their existing communication skills. These are valuable findings as such. However, at this stage we cannot conclude that PatientTIME improves patients’ confidence when interacting with health professionals. By integrating a process evaluation and an outcome evaluation in this way, we were able to demonstrate which elements of PatientTIME were used and appreciated, even though they did not lead to measurable changes in communication self-efficacy.

### Future Research

We identified three themes for future research. First, when evaluating the effectiveness of stand-alone Web-based interventions like PatientTIME, it may be worthwhile to consider research methods that allow the intervention to be dynamic, rather than freezing it in a certain state. In practice, Web-based interventions also need to be optimized continuously. With a more dynamic approach, intermediate test results can be used to improve the intervention and new versions of the intervention can be released during the evaluation phase. Second, when evaluating stand-alone interventions in a trial setting, the involvement of HCPs as informants might be crucial as a means of reaching a more diverse group of patients. More than half of the intervention group patients explained that the intervention helped them in the preparation for a clinical consultation, created awareness, and/or reinforced their current communication skills. Patients who reported no benefit explained that they already had a good understanding with their HCP, had a lot of experience, or considered themselves as sufficiently participative and empowered. Finally, it will be interesting to continue monitoring the use and effectiveness of PatientTIME once it is publicly available in order to provide input for other interventions and to keep PatientTIME up-to-date.

## Abbreviations

HCP: health care professional
MRC: Medical Research Council
PEPPI: Perceived Efficacy in Patient-Physician Interactions
QPS: question prompt sheet
RCT: randomized controlled trial
